# Mechanical Valve Thrombosis in a Pregnant Patient: A Case of Therapeutic Failure

**DOI:** 10.7759/cureus.5615

**Published:** 2019-09-10

**Authors:** Rahul Gupta, Purva Ranchal, Joseph Harburger

**Affiliations:** 1 Internal Medicine, Westchester Medical Center, Valhalla, USA; 2 Cardiology, Westchester Medical Center, Valhalla, USA

**Keywords:** mechanical heart valve, valve thrombosis, pregnancy, anticoagulation

## Abstract

Mechanical valve thrombosis is life-threatening complication especially in pregnant patients. The optimal anticoagulation regimen is still not certain as there are different fetal and maternal risks associated with anticoagulation.

A 37-year-old woman with a history of rheumatic heart disease with a mechanical mitral valve replacement 13 years prior presented to the hospital with dyspnea on mild exertion associated with orthopnea for three days. She was nine weeks pregnant, she had been on warfarin prior to pregnancy, and was switched to low molecular weight heparin (LMWH) in her 6th week of pregnancy. Fluoroscopy showed that one leaflet of the mitral valve was nearly fixed, while the other leaflet had restricted motion at maximal opening. Transesophageal echocardiogram (TEE) showed a very large thrombus approximately 3-4 cm^2^ encompassing the entire mechanical valve with one immobile leaflet and limited mobility in other leaflet. In view of her clinical status (dyspnea with NYHA Class IV symptoms), the patient underwent uncomplicated bioprosthetic mitral valve replacement. However, the fetus did not survive.

Mechanical heart valve (MHV) thrombosis is life-threatening complication in pregnancy. The optimal anticoagulation therapy in pregnancy is unclear. This case report brings into light that in spite of adequate anticoagulation, pregnant patients with mechanical heart valves are still at a high risk of developing valve thrombosis. It highlights the use of transthoracic and transesophageal echocardiogram along with fluoroscopy in diagnosing this and discusses the therapeutic options for this unique condition.

## Introduction

Mechanical valve thrombosis is a rare and potentially life-threatening complication, especially in patients with a pro-thrombotic state such as pregnancy [1]. Maintaining adequate anticoagulation is essential to reduce the risk of thromboembolic events. Due to different maternal and fetal risks associated with anticoagulation, along with absence of randomized prospective controlled trials, the optimal anticoagulation regimen in pregnant women remains uncertain [2, 3]. We present a case of mechanical mitral valve thrombosis in a pregnant female while on therapeutic anticoagulation.

## Case presentation

**Figure 1:**
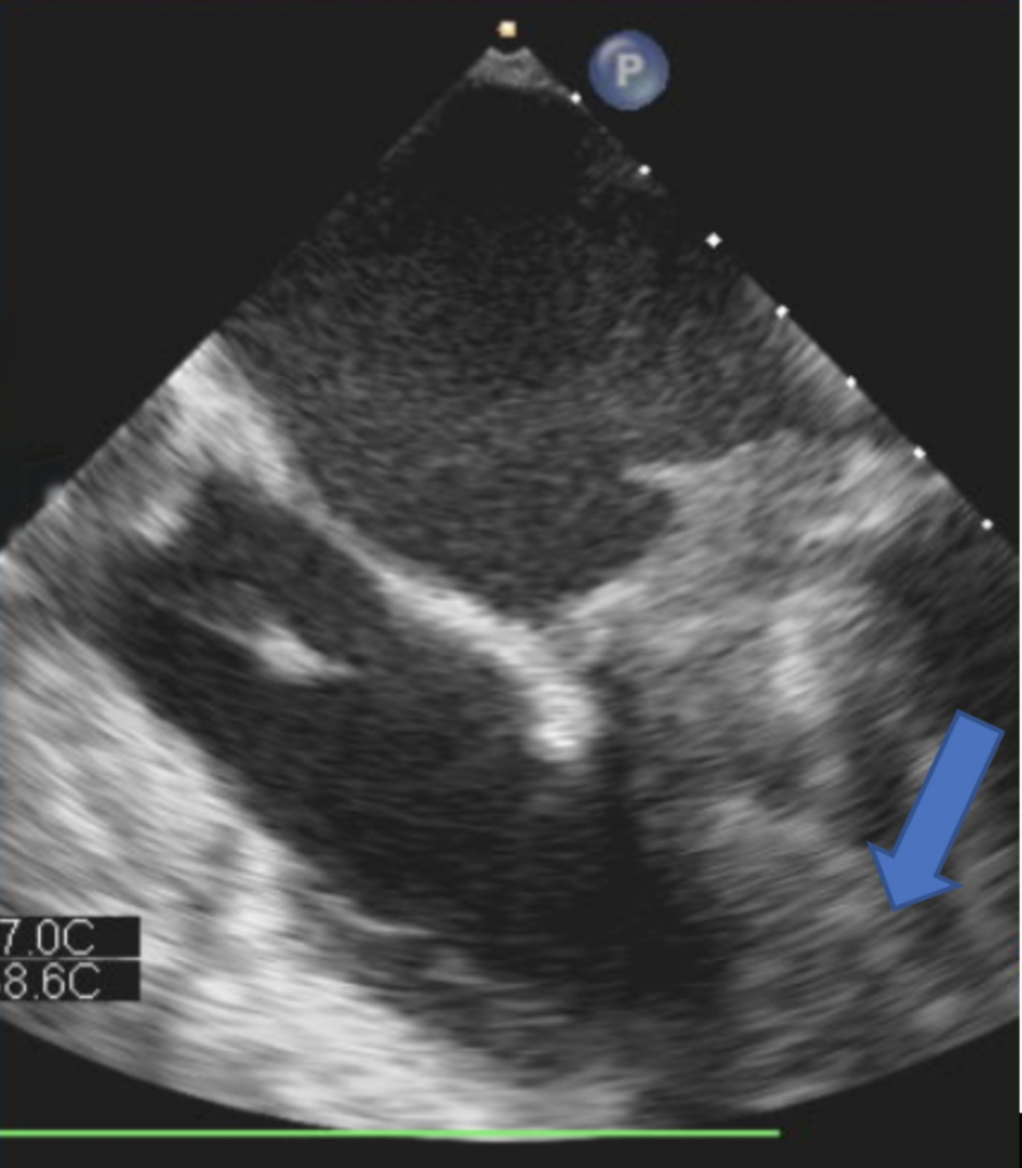
X-plane image of transesophageal echocardiography showing thrombus (Arrow) caked onto the mechanical mitral valve

**Figure 2:**
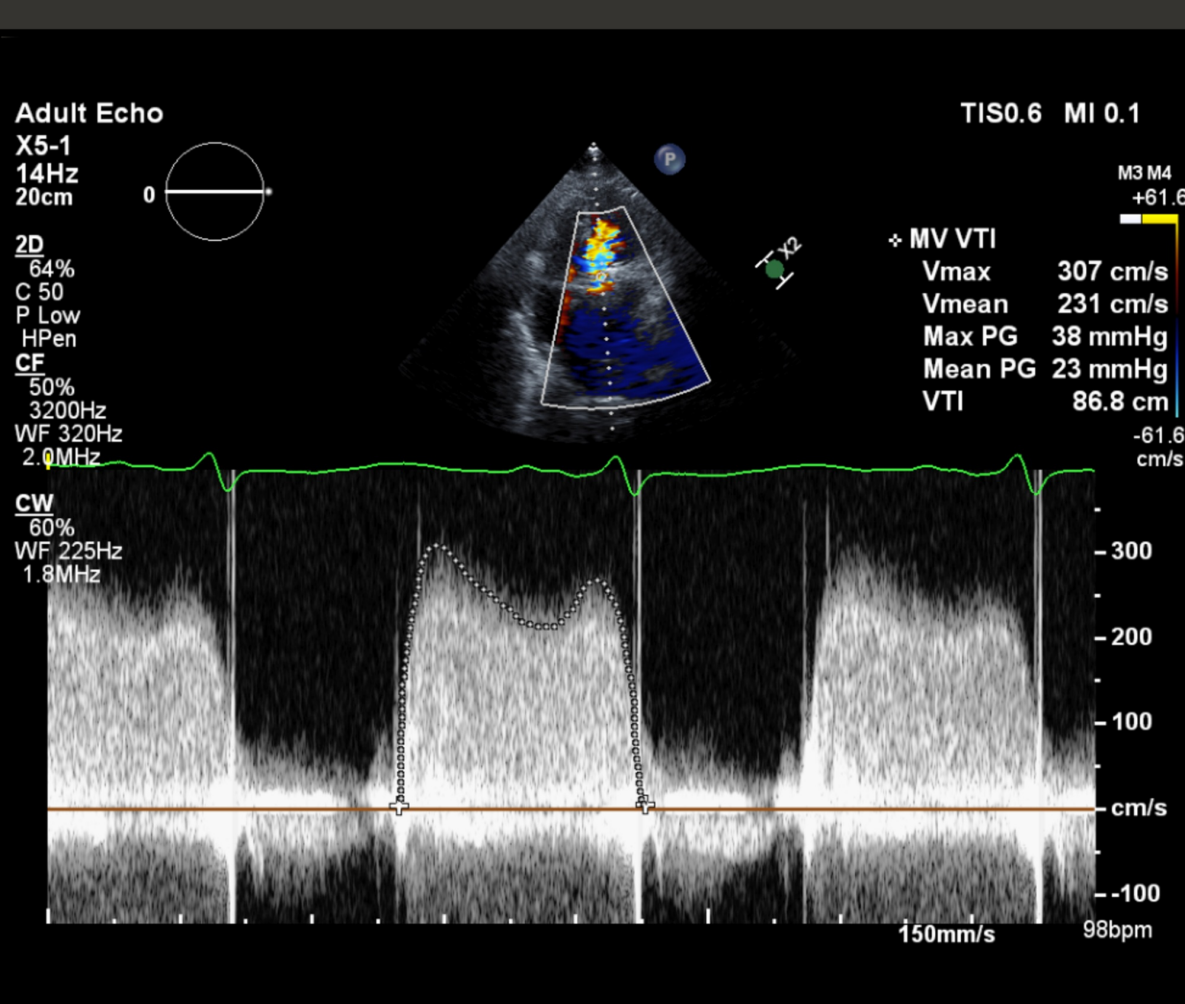
Transthoracic echocardiography doppler measurements showing increased mean pressure gradient across mechanical mitral valve

**Figure 3:**
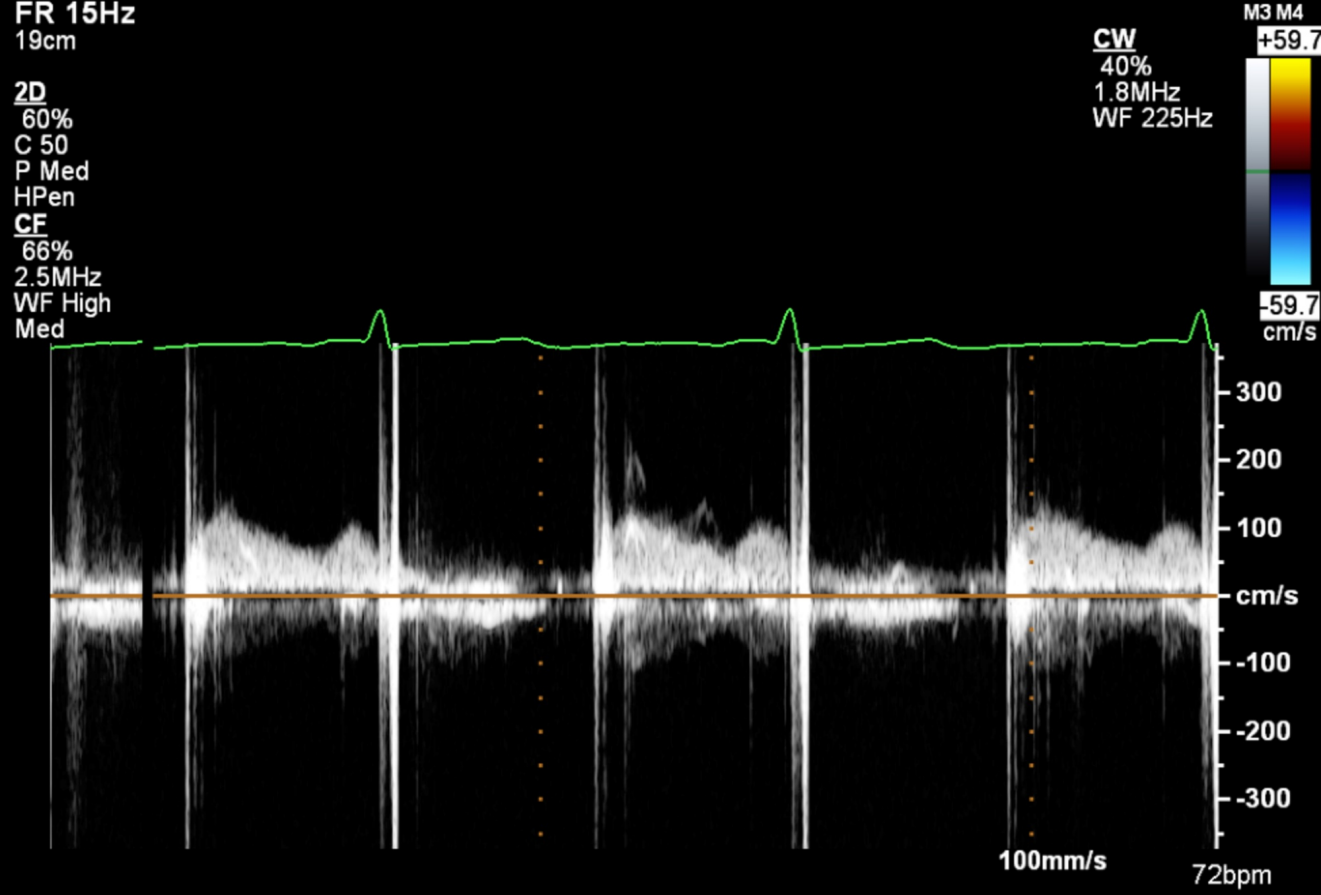
Transthoracic echocardiography doppler measurements showing normal mean pressure gradient across mechanical mitral valve prior to pregnancy

A 37-year-old woman with a history of rheumatic heart disease with a mechanical mitral valve replacement 13 years prior presented to the hospital with dyspnea on mild exertion associated with orthopnea for three days. She was nine weeks pregnant, she had been on warfarin 5.5 mg/day prior to pregnancy with therapeutic international normalized ratio (INR) levels, and was switched to low molecular weight heparin (LMWH) in her 6th week of pregnancy. Her anti-Xa levels were found to be in the therapeutic range (1.2 IU/ml). Physical exam was pertinent for mild bibasilar rales, with no jugular venous distension or pedal edema. Laboratory findings were significant for hemoglobin of 7.1 mg/dl, troponin level of 0.06 ng/ml with brain natriuretic peptide 252 pg/ml. Chest X-ray showed mild pulmonary vascular congestion. Transthoracic echocardiogram (TTE) showed a normal left ventricle with ejection fraction of 70%, a dilated left atrium, and a bileaflet mechanical mitral valve, with mean transmitral diastolic gradient of 23 mm Hg at heart rate of 98, peak mitral diastolic velocity 266 cm/sec, mitral pressure half time 235 ms, and mitral valve velocity time integral/left ventricle outflow tract velocity time integral ratio of 4.8, with findings highly suspicious for mechanical mitral valve stenosis (Figure 1). Prior to her pregnancy, she had a normal mean gradient of 4 mm Hg at heart rate of 72 (Figure 2). Fluoroscopy showed that one leaflet of the mitral valve was nearly fixed, while the other leaflet had restricted motion at maximal opening (Video 1). Trans-esophageal echocardiogram (TEE) showed a very large thrombus approximately 3-4 cm^2^ encompassing the entire mechanical valve with one immobile leaflet and limited mobility in other leaflet (Videos 2-4, Figure 3). Intravenous infusion of unfractionated heparin (UFH) was immediately initiated to maintain an activated partial thromboplastin time (APTT) 1.5 to 2 times higher than the upper normal limit. In view of her clinical status (dyspnea with NYHA Class IV symptoms) urgent surgical consultation was requested.

**Video 1:** Fluoroscopy showing fixed mechanical mitral valve leaflet with restricted mobility of the other leaflet during opening and closing of the valve

**Video 2:** Transesophageal echocardiography showing thrombus over the mechanical mitral valve

**Video 3:** Transesophageal echocardiography showing thrombus over the mechanical mitral valve

**Video 4:** Transesophageal echocardiography showing turbulent flow through the mechanical mitral valve due to the thrombus

After multidisciplinary evaluation and extensive discussions with the patient, a decision was made to perform a bioprosthetic mitral valve replacement. The patient had an uncomplicated bioprosthetic mitral valve replacement. However, the patient had missed abortion, most likely secondary to impact on placental perfusion secondary to down regulation of her blood volume and pressure, and her need for blood transfusion.

## Discussion

Anticoagulation in patients with mechanical heart valves (MHVs) during pregnancy is a challenging problem. The annual risk of a thrombotic event in patients not taking anticoagulation is approximately 4% while the risk in those on appropriate anticoagulation is 1% [4]. Oral vitamin K antagonists are the most effective anticoagulation regimen in patients with mechanical heart valves. However, vitamin K antagonists cross the placental barrier and can cause teratogenic effects, with reported incidence of teratogenicity between 0.6%-10% [5]. UFH and LMWH do not cross the placenta and do not have teratogenic effects. Current guidelines of the American College of Cardiology recommend continuing warfarin throughout the pregnancy if the dose is less than 5 mg/day [6, 7]. However, if the dose of warfarin exceeds 5 mg/day, strong consideration should be made to switch to UFH or LMWH to avoid teratogenicity. Dosing of UFH is done to maintain APTT 1.5-2 times control, while LMWH is dosed to maintain peak anti-Xa level measured four to six hours after administration of 1.0 IU/ml to 1.2 IU/ml.

Risk factors for prosthetic valve thrombosis include patients with old or small mechanical mitral valve, previous thromboembolic complications, multiple mechanical heart valves or presence of atrial fibrillation or flutter. Most cases of mechanical heart valve thrombosis in pregnant patients treated with LMWH have been due to inadequate dosing, lack of monitoring, or subtherapeutic anti-Xa levels [8]. Our patient developed mechanical valve thrombosis despite adequate LMWH dosing and monitoring.

Therapeutic options for mechanical valve thrombosis include medical therapy versus surgical replacement of the valve. Valve repair or replacement in pregnancy is indicated in pregnancy patients who remain symptomatic even after medical therapy [9]. Recently published guidelines (“2017 ESC/EACTS Guidelines for the management of valvular heart disease”) recommend surgical management for obstructive mechanical valve thromboses when there is no contraindication to surgery [10]. Our patient had NYHA class IV symptoms and large thrombotic burden warranting urgent surgical management. The increased mortality and morbidity associated with valve thrombosis in pregnant patients warrants extensive pre-pregnancy counseling and centralization of care, which could result in better outcomes in future.

## Conclusions

Thrombotic complications of MHV mostly occur during the first trimester when anticoagulant regimens are switched from one regimen to another. The use of LMWH warrants monitoring and appropriate dose adjustments to maintain adequate anti-factor Xa levels to decrease the incidence of thromboembolic complications. In spite of that, there is still a risk of developing valve thrombosis as seen in our patient. The increased mortality and morbidity associated with mechanical valve thrombosis in pregnancy warrants extensive pre-pregnancy counseling and centralization of care.
